# Expression of the multidrug resistance-associated protein (MRP) gene in colorectal carcinomas.

**DOI:** 10.1038/bjc.1997.35

**Published:** 1997

**Authors:** M. Fillpits, R. W. Suchomel, G. Dekan, W. Stiglbauer, K. Haider, D. Depisch, R. Pirker

**Affiliations:** Department of Oncology, University of Vienna Medical School, Austria.

## Abstract

To determine the clinical significance of MRP in patients with colorectal carcinomas, we have studied the expression of the MRP gene by reverse transcription-polymerase chain reaction (RT-PCR) (n = 105) and by immunohistochemistry (n = 30). MRP mRNA expression was observed in 92 (88%) tumour specimens. Positive MRP staining with monoclonal antibodies QCRL-1 and QCRL-3 was detected in all samples studied with strong staining in seven (23%) and weak staining in 23 (77%) specimens. Strong MRP staining in these samples did not appear to be related to the age and sex of the patients, localization of the primary tumour, histological grade, tumour size, lymph node metastasis, distant metastasis and tumour stage. Strong MRP staining was not associated with MDR1 RNA or P-glycoprotein (P-gp) expression. Kaplan-Meier curves revealed that overall survival of patients with strong MRP-staining tumours was similar to the survival of patients with weak-staining tumours. These data indicate that the MRP gene is expressed in primary colorectal carcinomas but is neither related to known prognostic factors nor a prognostic factor by itself.


British Journal of Cancer (1997) 75(2), 208–212
© 1997 Cancer Research Campaign

### Expression of the multidrug resistance-associated protein (*MRP*) gene in colorectal carcinomas

**M Filipits[1], RW Suchomel[1], G Dekan[2], W Stiglbauer[3], K Haider[4], D Depisch[4] and R Pirker[1]**

[1]Department of Oncology, Clinic for Internal Medicine I and[2] Department of Pathology, University of Vienna Medical School, A-1090 Vienna, Austria; Departments of [3]Pathology and [4]Surgery, General Hospital Wiener Neustadt, A-2700 Wiener Neustadt, Austria

**Summary** To determine the clinical significance of MRP in patients with colorectal carcinomas, we have studied the expression of the *MRP* gene by reverse transcription–polymerase chain reaction (RT–PCR) ($n$=105) and by immunohistochemistry ($n$=30). *MRP* mRNA expression was observed in 92 (88%) tumour specimens. Positive MRP staining with monoclonal antibodies QCRL-1 and QCRL-3 was detected in all samples studied with strong staining in seven (23%) and weak staining in 23 (77%) specimens. Strong MRP staining in these samples did not appear to be related to the age and sex of the patients, localization of the primary tumour, histological grade, tumour size, lymph node metastasis, distant metastasis and tumour stage. Strong MRP staining was not associated with *MDR1* RNA or P-glycoprotein (P-gp) expression. Kaplan–Meier curves revealed that overall survival of patients with strong MRP-staining tumours was similar to the survival of patients with weak-staining tumours. These data indicate that the *MRP* gene is expressed in primary colorectal carcinomas but is neither related to known prognostic factors nor a prognostic factor by itself.

**Keywords:** colorectal carcinoma; multidrug resistance; *MRP* gene; multidrug resistance-associated protein

Drug resistance remains a major problem in patients with colorectal carcinomas, which are *usually* intrinsically resistant to anti-cancer drugs. The clinically relevant mechanisms of drug resistance are currently under investigation. One important mechanism may be the multidrug-resistance phenotype (Pastan and Gottesman, 1987; Gottesman and Pastan, 1988; Simon and Schindler, 1994). Multidrug resistance (MDR) is the term for the resistance against a variety of hydrophobic natural compounds, including several anti-cancer drugs (Pastan and Gottesman, 1987).

MDR is caused in part by overexpression of the *MDR1* gene (Goldstein et al, 1989). This gene codes for P-glycoprotein (P-gp), a 170-kDa transmembrane protein, which may function as an energy-dependent drug efflux pump (Pastan and Gottesman, 1987; Gottesman and Pastan, 1988). Besides the ‘active drug pump model’, the ‘altered partitioning model’ is discussed. The latter model proposes that altered sequestration of anti-cancer drugs and other compounds is the indirect result of perturbations in the character and/or magnitude of eukaryotic plasma membrane electrochemical potential caused by P-gp overexpression (Roepe, 1995). Expression of *MDR1* mRNA and P-gp was demonstrated in 65% and 68% of primary colorectal carcinomas respectively (Weinstein et al, 1991; Pirker et al, 1993). Although MDR was previously thought to be predominantly caused by the expression of the *MDR1* gene, it is now increasingly believed to be caused by other mechanisms also (Roepe et al, 1993; Simon and Schindler, 1994; Filipits et al, 1996*a*).

*Received 24 June 1996*
*Revised 14 August 1996*
*Accepted 21 August 1996*

*Correspondence to:* R Pirker, Department of Oncology, Clinic for Internal Medicine I, University of Vienna Medical School, Währinger Gürtel 18–20, A-1090 Vienna, Austria

Recently, overexpression of the multidrug resistance-associated protein (MRP) was suggested as a possible mechanism for non-P-gp-mediated MDR (Cole et al, 1992; Barrand et al, 1994). This 190-kDa membrane protein is encoded by the *MRP* gene, which has recently been cloned (Cole et al, 1992). Transfection studies have demonstrated that overexpression of human MRP confers a multidrug-resistance phenotype (Grant et al, 1994; Kruh et al, 1994; Zaman et al, 1994). MRP is believed to be involved in the ATP-dependent transport of cysteinyl leukotrienes (e.g. $LTC_4$) and other glutathione-*S*-conjugates (Loe et al, 1996). MRP has also been suggested to be involved in the transport of vincristine in the presence of reduced glutathione (Loe et al, 1996). However, the exact mechanisms by which MRP mediates resistance to anti-cancer drugs remains to be determined.

The aim of our present study was, firstly, to determine the expression of the *MRP* gene in primary colorectal carcinomas in order to evaluate the clinically active mechanisms of MDR in these tumours further, and, secondly, to assess its association with other clinical parameters, including the survival of patients.

### PATIENTS AND METHODS

### Patients

Between 1988 and 1995, 105 patients with colorectal carcinomas were admitted to this study. Sixty-five patients had been included in a previous study (Pirker et al, 1993). The patients were treated at the Surgical Department of the General Hospital of Wiener Neustadt, Austria.

Forty-two patients received adjuvant chemotherapy with 5-fluorouracil and leucovorin as described (Pirker et al, 1993). During metastatic disease, patients were usually treated with 5-fluorouracil with or without leucovorin and occasionally also with cisplatin and drugs to which P-gp overexpression may confer resistance (mitoxantrone and mitomycin C).

### Tumour specimens and cell lines

Colorectal carcinoma specimens and adjacent normal tissue specimens were obtained by surgery and stored at –80°C until use. Samples were graded for histological type and Dukes' stage according to standard criteria. Peripheral blood mononuclear cells were obtained from healthy volunteers by Ficoll–Paque gradient centrifugation. KB-3-1 and KB-8-5 cells (provided by Drs I Pastan and M Gottesman, National Cancer Institute, Bethesda, MD, USA) were grown as described (Pirker et al, 1991). Cytospins of C1 and T5 cells as well as cDNAs synthesized from RNA that had been isolated from these cells were kindly provided by Drs S Cole and R Deeley (Queen's University, Kingston, Canada).

### Analysis of gene expression by RT–PCR

Total RNA was extracted from tumour specimens by means of RNAzol (Cinna Scientific, Cincinnati, OH, USA) and quantitated spectrophotometrically. *MRP* RNA was determined as described recently (Filipits et al, 1996*b*).

Briefly, 5–10 μg of total RNA was used for cDNA synthesis using Moloney murine leukaemia virus reverse transcriptase (Promega) in a total volume of 50 μl. After 1 h incubation at 37°C, 5 min at 95°C and a quick chill to 4°C, cDNA was stored at -20°C until use.

The cDNA reaction mixture (5–10 μl) was used for amplification of specific DNA sequences. A total of 30–35 cycles at 95°C for 25 s, 57°C for 30 s and 73°C for 1 min and a quick chill to 4°C in a 9600 thermocycler (Perkin Elmer Cetus, Emeryville, CA, USA) were performed. Samples, which were *MRP* mRNA negative after 30 cycles, were re-evaluated with 35 cycles under otherwise unchanged conditions. In this study, all oligonucleotides used as primers were synthesized by Fa. Biomedica (Vienna, Austria). The primers were chosen as follows: 5′-TGAAGGACTTCGTGTCAGCC-3′ (forward primer; residues 4419–4438) and 5′-GTCCATGATGGTGTTGAGCC-3′ (reverse primer; residues 4656–4675) of the *MRP* gene (Zaman et al, 1993), 5′-ACCCCCACTGAAAAAGATGA-3′ (forward primer; residues 1544–1563) and 5′-ATCTTCAAACCTCCATGATG-3′ (reverse primer; residues 2253–2262 and 3508–3517) of the $\beta_2$-microglobulin ($\beta_2$-*m*) gene (Noonan et al, 1990), and 5′-CCCATCATTGCAATAGCAGG-3′ (forward primer; residues 2596–2615) and 5′-GTTCAAACTTCTGCTCCTGA-3′ (reverse primer; residues 2733–2752) of the *MDR1* gene (Noonan et al, 1990). All corresponding pairs of the primers spanned an intron to control against contamination by amplification of genomic DNA sequences. The sizes of the PCR products are 256 bp (*MRP*), 120 bp ($\beta_2$-*m*) and 167 bp (*MDR1*) respectively. Expression of $\beta_2$-*m* RNA was used as an internal control for both *MDR1* and *MRP* gene expression.

### Immunohistochemical analysis

Monoclonal antibodies QCRL-1 and QCRL-3 (kindly provided by Drs S Cole and R Deeley, Queen's University, Kingston, Canada), which recognize different epitopes of MRP (Hipfner et al, 1994), as well as monoclonal antibodies C219 and MRK16, which recognize different epitopes of P-gp, were used for immunohistochemistry as described previously (Filipits et al, 1996*b*).

Cryostat sections (4 μm) of the colorectal carcinoma specimens were prepared. Serial sections were used for MRP and P-gp staining. The slides were air dried overnight and fixed in cold acetone (QCRL-1, QCRL-3 and C219) or paraformaldehyde (MRK16). After blocking of endogenous peroxidase activity, the slides were incubated with normal goat serum followed by 2 h incubation with the primary antibodies. Antibody binding was detected by the avidin–biotin–peroxidase method. Negative controls were performed without the primary antibodies for each sample and, in addition, with irrelevant isotype-matched antibodies in some cases. C1 and T5 cells were used as negative and positive controls for MRP expression (Grant et al, 1994). Drug-sensitive KB-3-1 and multidrug-resistant KB-8-5 cells served as negative and positive controls for P-gp expression respectively.

**Table 1** *MRP* and *MDR1* gene expression in colorectal carcinoma specimens

**(a) RT-PCR**

| | Negative (%) | Positive (%) |
|---|---|---|
| *MRP* RNA (*n*=105) | 13 (12) | 92 (88) |
| *MDR1* RNA (*n*=100) | 17 (17) | 83 (83) |

**(b) Immunohistochemistry**

| | Negative (5) | Weak (%) | Strong (%) |
|---|---|---|---|
| MRP (*n*=30) | 0 ( 0) | 23 (77) | 7 (23) |
| P-gp | | | |
| C219 (*n*=30) | 18 (60) | 8 (27) | 4 (13) |
| MRK16 (*n*=30) | 16 (53) | 9 (30) | 5 (17) |

Immunostained slides were independently examined by two observers who had no previous knowledge of the clinical outcome of the patients. MRP and P-gp immunostaining were evaluated and scored separately.

### Survival analysis

Durations of overall survival (OS) were estimated according to Kaplan and Meier (1958). OS was measured from the time of diagnosis until the time of death or in the case of censored patients until the time of the last control.

### Statistical analysis

Frequencies were examined by chi-square tests. In addition, Kruskal–Wallis tests were performed. Survival curves were compared by the Wilcoxon test.

### RESULTS

### MRP expression in colorectal carcinoma specimens

*MRP* mRNA expression of primary colorectal carcinoma specimens (*n*=105) was determined by RT–PCR. Drug-sensitive C1 and drug-resistant T5 cells were used as negative and positive controls respectively. In addition, peripheral blood mononuclear cells served as positive controls. The $\beta_2$-*m* gene, which was coamplified with the *MRP* gene, was used as an internal control. Ninety-two (88%) colorectal carcinomas did express *MRP* mRNA (Table 1). Thirteen carcinomas were negative for *MRP* mRNA (Table 1) and remained negative when the number of RT–PCR cycles was raised

© Cancer Research Campaign 1997

Table 2 MRP and clinical parameters of the patients

| | All patients | Patients with weak MRP staining | Patients with strong MRP staining | P-value |
|---|---|---|---|---|
| Number of patients | 30 | 23 ( 77) | 7 (23) | |
| Age (years) | | | | |
| Median | 65 | 66 | 60 | NS |
| Range | 49–81 | 49–81 | 49–73 | |
| Sex (F/M) | 10/20 | 8/15 | 2/5 | NS |
| Localization | | | | |
| Colon | 16 | 12 ( 75) | 4 (25) | NS |
| Rectum | 14 | 11 ( 79) | 3 (21) | |
| Histological grade | | | | |
| G0–1 | 6 | 3 ( 50) | 3 (50) | NS |
| G2–3 | 24 | 20 ( 83) | 4 (17) | |
| Primary tumour | | | | |
| T1–2 | 7 | 5 ( 71) | 2 (29) | NS |
| T3–4 | 23 | 18 ( 78) | 5 (22) | |
| Regional lymph nodes | | | | |
| N0 | 13 | 9 ( 69) | 4 (31) | |
| N1 | 12 | 10 ( 83) | 2 (17) | NS |
| N2 | 3 | 2 ( 67) | 1 (33) | |
| N3 | 2 | 2 (100) | 0 ( 0) | |
| Distant metastasis | | | | |
| M0 | 28 | 21 ( 75) | 7 (25) | NS |
| M1 | 2 | 2 (100) | 0 ( 0) | |
| Tumour stage (Dukes' stage) | | | | |
| A2 | 3 | 2 ( 67) | 1 (33) | |
| B | 10 | 7 ( 70) | 3 (30) | |
| C1 | 10 | 9 ( 90) | 1 (10) | NS |
| C2 | 5 | 3 ( 60) | 2 (40) | |
| D | 2 | 2 (100) | 0 ( 0) | |
| *MDR1* | | | | |
| *MDR1* RNA positive | 24 | 19 ( 79) | 5 (21) | |
| P-gp (C219) positive | 12 | 9 ( 75) | 3 (25) | NS |
| P-gp (MRK16) positive | 14 | 9 ( 64) | 5 (36) | |
| Treated with chemotherapy | 18 | 15 ( 83) | 3 (17) | NS |
| Treated with MDR drugs | 7 | 5 ( 71) | 2 (29) | NS |

MRP expression of colorectal carcinoma specimens was determined by immunohistochemistry and correlated with clinical parameters of the patients. Statistical analysis was performed by either Kruskal–Wallis or chi-square test. Numbers in parentheses are percentages. NS, not significant.

from 30 to 35 (data not shown).

### Immunohistochemistry

MRP expression at the protein level was immunohistochemically determined by means of monoclonal antibodies, QCRL-1 and QCRL-3, on frozen sections of colorectal carcinomas and, in some cases, also on adjacent normal tissue specimens. Non-specific binding was excluded by controls either with irrelevant isotype-matched monoclonal antibodies ($IgG_1$ and $IgG_{2A}$) or without primary antibodies.

Immunohistochemical analysis was performed in 30 out of the 105 colorectal carcinoma specimens studied by RT–PCR. All of the 30 specimens showed detectable levels of *MRP* RNA. Both plasma membrane and cytoplasmatic MRP staining patterns were seen. With regard to the degree of MRP expression, patients were divided according to the intensity of staining into a group with strong and a second group with only weak staining. Staining of the positive control cell line T5 was chosen as very strong and above the intensity seen in clinical samples. Staining with anti-MRP antibodies was strong in seven (23%) specimens and only weak in the remaining 23 (77%) samples (Table 1). In the case of strong staining, the majority of tumour cells within the specimens were affected. Completely negative MRP staining was not seen in any of the carcinoma specimens. Unfortunately, no samples from RT–PCR-negative tumours were available for immunohistochemistry.

In addition, MRP expression of normal colon tissue adjacent to the carcinomas was assessed in some samples. Whereas MRP staining could be detected in all normal colon tissues, strong MRP staining occurred predominantly on the luminal surface of crypt epithelial cells.

### Relationship between the *MRP* gene and the *MDR1* gene

In order to assess the relationship between the *MRP* and the *MDR1* gene, we compared the expression of both genes. *MDR1* RNA was

 © Cancer Research Campaign 1997

**Figure 1** MRP expression and overall survival. Overall survival (OS) of the patients with weak ($n$=23) or strong MRP staining ($n$=7) was calculated according to Kaplan and Meier (1958). Statistical comparison between the curves was done by the Wilcoxon test determined by RT–PCR and was detected in 83 (83%) samples (Table 1). P-gp expression was also studied immunohistochemically by means of the monoclonal antibodies C219 and MRK16 respectively, and was found to be positive in 40% (13% strong staining) and 47% (17% strong staining) of the tumour specimens respectively (Table 1). No significant correlation between MRP and *MDR1* mRNA or P-gp expression was observed (Table 2).

### MRP in relation to clinical parameters

Next, we evaluated the association of *MRP* gene expression with clinical parameters. Histological examination revealed adenocarcinomas in all cases (data not shown). *MRP* gene expression of the tumours did not appear to be related to sex and age of the patients, localization and size of the primary tumour, histological grade, tumour infiltration of the lymph nodes, distant metastasis and tumour stage (Table 2).

To evaluate whether strong MRP staining of the tumours is of prognostic value, Kaplan–Meier analysis of overall survival was performed in 30 patients. At a median follow-up of 39 months, overall survival of patients with weak-staining tumours was similar to the survival of patients with strong-staining tumours (Figure 1).

### DISCUSSION

In the present study, expression of *MRP* RNA was detected in 88% of the primary colorectal carcinomas. MRP, as determined by immunohistochemistry, was expressed in all tumour specimens, with strong staining in 23% of the samples. These findings suggest that the *MRP* gene might contribute to the intrinsic drug resistance of colorectal carcinomas. Previously, expression of the *MDR1* gene, which was seen in approximately two out of three primary colorectal carcinomas, was thought to be the predominant mechanism of MDR in these tumours (Goldstein et al, 1989; Weinstein et al, 1991; Pirker et al, 1993; Sinicrope et al, 1994). Our percentage of strong MRP-staining tumours is consistent with the recent report on MRP expression in four out of 12 (33%) colorectal carcinomas (Nooter et al, 1995). The percentage of P-gp-positive tumours in our study (40% and 47%) is also similar to the percentages previously reported (Weinstein et al, 1991; Mayer et al, 1993; De Angelis et al, 1995).

Strong MRP staining did not appear to be related to size of the primary tumour, lymph node involvement, distant metastasis, tumour stage and survival of the patients (Table 2). Thus, MRP was not related to established prognostic factors and was not of prognostic value by itself. Lack of an association of *MRP* gene expression and clinical outcome of the patients can be explained by several reasons. Firstly, the clinical behaviour of the tumours is independent of MRP expression. In lung tumours, *MRP* RNA expression was suggested to be involved in invasion because it was more prominent in cells at the leading edge of the tumours, but no data on its relation to the survival of the patients are reported (Thomas et al, 1994). Secondly, a functionally active *MRP* gene was most likely without clinical impact because most patients were not treated with MDR drugs and because the activity of 5-fluorouracil is not affected by MRP. Finally, a type II statistical error cannot be excluded. Therefore, it might be worthwile to confirm these results in a larger study population.

In one report, P-gp expression was observed predominantly in invasively growing tumour cells, suggesting that P-gp expression is associated with local tumour aggressiveness (Weinstein et al, 1991). Despite these findings however, neither *MDR1* mRNA nor P-gp expression of the tumours was of prognostic value in patients with colorectal carcinomas (Mayer et al, 1993; Pirker et al, 1993).

No correlation between *MRP* gene expression and *MDR1* gene expression was observed (Table 2). In contrast to these results, sequential coexpression of the *MRP* and the *MDR1* gene was detected in etoposide-selected H69 small-cell lung cancer cells (Brock et al, 1995).

Recently, one mutant p53 has been shown to stimulate the *MDR1* promoter *in vitro,* whereas wild-type p53 represses its activity (Chin et al, 1992). In colorectal carcinoma specimens, however, P-gp expression was independent of p53 expression or the incidence of p53 mutations (De Angelis et al, 1995), suggesting that mutant p53 does not induce overexpression of P-gp in colorectal carcinomas. *MRP* gene expression correlated with amplification and overexpression of the *n-myc* oncogene in childhood neuroblastoma (Bordow et al, 1994; Norris et al, 1996). Thus, future studies will have to define further the relationship between drug resistance genes and oncogenes or tumour-suppressor genes in colorectal carcinomas.

*MRP* gene expression could be involved in the clinically well-known intrinsic resistance of colorectal carcinomas to MDR drugs. Because in vitro MRP is believed to be involved in the transport of certain anti-cancer drugs, including anthracyclines and vinca alkaloids (Cole et al, 1994; Grant et al, 1994), it is anticipated that this function also contributes to the inactivity of these drugs in the treatment of colorectal carcinomas. Expression of MRP might also be one of the reasons why previous clinical trials with resistance modifiers of the *MDR1* gene (Pirker et al, 1990; Twentyman, 1992) failed in patients with colorectal carcinomas and other tumours (Lehnert, 1993; Milroy et al, 1993).

In conclusion, multidrug resistance in colorectal carcinomas is a complex phenomenon probably involving both the *MRP* gene and the *MDR1* gene. Future studies will have to address their regulation of expression and quantitative contribution to resistance to anti-cancer drugs. The presence of *MRP* gene expression will also have to be considered in the planning of future clinical trials with drug resistance modifiers. In addition, other mechanisms involved in the MDR of cell lines will also have to be studied in colorectal carcinomas (Beck, 1989; Simon and Schindler, 1994). Only knowledge of all clinically important mechanisms of drug

© Cancer Research Campaign 1997

resistance might eventually enable clinicians to design ways to overcome drug resistance and, thereby, improve the outcome of chemotherapy in patients with colorectal carcinomas.

### ACKNOWLEDGEMENTS

This study was supported by 'Jubiläumsfonds der Österreichischen Nationalbank' (Project number: 5260) and 'Kommission Onkologie der Medizinischen Fakultät der Universität Wien'.

### REFERENCES

Barrand MA, Heppell-Parton AC, Wright KA, Rabbitts PH and Twentyman PR (1994) A 190-kilodalton protein overexpressed in non-P-glycoprotein-containing multidrug-resistant cells and its relationship to the MRP gene. *J Natl Cancer Inst* **86**: 110–117

Beck WT (1989) Unknotting the complexities of multidrug resistance: the involvement of DNA topoisomerases in drug action and resistance. *J Natl Cancer Inst* **81**: 1683–1685

Bordow SB, Haber M, Madafiglio J, Cheung B, Marshall GM and Norris MD (1994) Expression of the multidrug resistance-associated protein (*MRP*) gene correlates with amplification and overexpression of the n-*myc* oncogene in childhood neuroblastoma. *Cancer Res* **54**: 5036–5040

Brock I, Hipfner DR, Nielsen BS, Jensen PB, Deeley RG and Cole SPC (1995) Sequential coexpression of the multidrug resistance genes *MRP* and *mdr1* and their products in VP-16 (etoposide)-selected H69 small cell lung cancer cells. *Cancer Res* **55**: 459–462

Chin K-V, Ueda K, Pastan I and Gottesman MM (1992) Modulation of activity of the promoter of the human *MDR1* gene by Ras and p53. *Science* **255**: 459–462

Cole SPC, Bhardwaj G, Gerlach JH, Mackie JE, Grant CE, Almquist KC, Stewart AJ, Kurz EU, Duncan AMV and Deeley RG (1992) Overexpression of a transporter gene in a multidrug-resistant human lung cancer cell line. *Science* **258**: 1650–1654

Cole SPC, Sparks KE, Fraser K, Loe DW, Grant CE, Wilson GM and Deeley RG (1994) Pharmacological characterization of multidrug resistant MRP-transfected human tumor cells. *Cancer Res* **54**: 5902–5910

De Angelis P, Stokke T, Smedshammer L, Lothe RA, Lehne G, Chen Y and Clausen OPF (1995) P-glycoprotein is not expressed in a majority of colorectal carcinomas and is not regulated by mutant p53 *in vivo*. *Br J Cancer* **72**: 307–311

Filipits M, Suchomel RW, Zöchbauer S, Malayeri R and Pirker R (1996*a*) Clinical relevance of drug resistance genes in malignant diseases. *Leukemia* **10** (suppl. 3): 10–17

Filipits M, Suchomel RW, Dekan G, Haider K, Valdimarsson G, Depisch D and Pirker R (1996*b*) *MRP* and *MDR1* gene expression in primary breast carcinomas. *Clin Cancer Res* **2**: 1231–1237

Goldstein LJ, Galski H, Fojo A, Willingham M, Lai S-L, Gazdar A, Pirker R, Green A, Crist W, Brodeur GM, Lieber M, Cossman J, Gottesman MM and Pastan I (1989) Expression of a multidrug resistance gene in human cancers. *J Natl Cancer Inst* **81**: 116–124

Gottesman MM and Pastan I (1988) The multidrug transporter, a double-edged sword. *J Biol Chem* **263**: 12163–12166

Grant CE, Valdimarsson G, Hipfner DR, Almquist KC, Cole SPC and Deeley RG (1994) Overexpression of multidrug resistance-associated protein (MRP) increases resistance to natural product drugs. *Cancer Res* **54**: 357–361

Hipfner DR, Gauldie SD, Deeley RG and Cole SPC (1994) Detection of the $M_r$ 190,000 multidrug resistance protein, MRP, with monoclonal antibodies. *Cancer Res* **54**: 5788–5792

Kaplan EL and Meier P (1958) Nonparametric estimation from incomplete observations. *J Am Stat Assoc* **53**: 457–481

Kruh GD, Chan A, Myers K, Gaughan K, Miki T and Aaronson SA (1994) Expression complementary DNA library transfer establishes *mrp* as a multidrug resistance gene. *Cancer Res* **54**: 1649–1652

Lehnert M (1993) Reversal of P-glycoprotein-associated multidrug resistance: the challenge continues. *Eur J Cancer* **29A**: 636–638

Loe DW, Almquist KC, Deeley RG and Cole SPC (1996) Multidrug resistance protein (MRP)-mediated transport of leukotriene $C_4$ and chemotherapeutic agents in membrane vesicles. *J Biol Chem* **271**: 9675–9682

Mayer A, Takimoto M, Fritz E, Schellander G, Kofler K and Ludwig H (1993) The prognostic significance of proliferating cell nuclear antigen, epidermal growth factor receptor, and *mdr* gene expression in colorectal cancer. *Cancer* **71**: 2454–2460

Milroy R on behalf of the West of Scotland Lung Cancer Research Group and the Aberdeen Oncology Group (1993) A randomised clinical study of verapamil in addition to combination chemotherapy in small cell lung cancer. *Br J Cancer* **68**: 813–818

Noonan KE, Beck C, Holzmayer TA, Chin JE, Wunder JS, Andrulis IL, Gazdar AF, Willman CL, Griffith B, Von Hoff DD and Roninson IB (1990) Quantitative analysis of *MDR1* (multidrug resistance) gene expression in human tumors by polymerase chain reaction. *Proc Natl Acad Sci USA* **87**: 7160–7164

Nooter K, Westerman AM, Flens MJ, Zaman GJR, Scheper RJ, Van Wingerden KE, Burger H, Oostrum R, Boersma T, Sonneveld P, Gratama JW, Kok T, Eggermont AMM, Bosman FT and Stoter G (1995) Expression of the multidrug resistance-associated protein (*MRP*) gene in human cancers. *Clin Cancer Res* **1**: 1301–1310

Norris MD, Bordow SB, Marshall GM, Haber PS, Cohn SL and Haber M (1996) Expression of the gene for multidrug-resistance-associated protein and outcome in patients with neuroblastoma. *N Engl J Med* **334**: 231–238

Pastan I and Gottesman M (1987) Mutliple-drug resistance in human cancer. *N Engl J Med* **316**: 1388–1393

Pirker R, Keilhauer G, Raschack M, Lechner C and Ludwig H (1990) Reversal of multi-drug resistance in human KB cell lines by structural analogs of verapamil. *Int J Cancer* **45**: 916–919

Pirker R, Wallner J, Geissler K, Linkesch W, Haas OA, Bettelheim P, Hopfner M, Scherrer R, Valent P, Havelec L, Ludwig H and Lechner K (1991) MDR1 gene expression and treatment outcome in acute myeloid leukemia. *J Natl Cancer Inst* **83**: 708–712

Pirker R, Wallner J, Gsur A, Götzl M, Zöchbauer S, Scheithauer W and Depisch D (1993) MDR1 gene expression in primary colorectal carcinomas. *Br J Cancer* **68**: 691–694

Roepe PD (1995) The role of the MDR protein in altered drug translocation across tumor cell membranes. *Biochim Biophys Acta* **1241**: 385–406

Roepe PD, Wei LY, Cruz J and Carlson D (1993) Lower electrical membrane potential and altered pH homeostasis in multidrug-resistant (MDR) cells: further characterization of a series of MDR cell lines expressing different levels of P-glycoprotein. *Biochemistry* **32**: 11042–11056

Simon SM and Schindler M (1994) Cell biological mechanisms of multidrug resistance in tumors. *Proc Natl Acad Sci USA* **91**: 3497–3504

Sinicrope FA, Hart J, Brasitus TA, Michelassi F, Lee JJ and Safa AR (1994) Relationship of P-glycoprotein and carcinoembryonic antigen expression in human colon carcinoma to local invasion, DNA ploidy, and disease relapse. *Cancer* **74**: 2908–2917

Thomas GA, Barrand MA, Stewart S, Rabbitts PH, Williams ED and Twentyman PR (1994) Expression of the multidrug resistance-associated protein (*MRP*) gene in human lung tumours and normal tissue as determined by *in situ* hybridisation. *Eur J Cancer* **30A**: 1705–1709

Twentyman PR (1992) MDR1 (P-glycoprotein) gene expression–implications for resistance modifier trials. *J Natl Cancer Inst* **84**: 1458–1460

Weinstein RS, Jakate SM, Dominguez JM, Lebovitz MD, Koukoulis GK, Kuszak JR, Klusens LF, Grogan TM, Saclarides TJ, Roninson IB and Coon JS (1991) Relationship of the expression of the multidrug resistance gene product (P-glycoprotein) in human colon carcinoma to local tumor aggressiveness and lymph node metastasis. *Cancer Res* **51**: 2720–2726

Zaman GJR, Versantvoort CHM, Smit JJM, Eijdems EWHM, De Haas M, Smith AJ, Broxterman HJ, Mulder NH, De Vries EGE, Baas F and Borst P (1993) Analysis of the expression of *MRP*, the gene for a new putative transmembrane drug transporter, in human multidrug resistant lung cancer cell lines. *Cancer Res* **53**: 1747–1750

Zaman GJR, Flens MJ, Van Leusden MR, De Haas M, Mulder HS, Lankelma J,

 © Cancer Research Campaign 1997


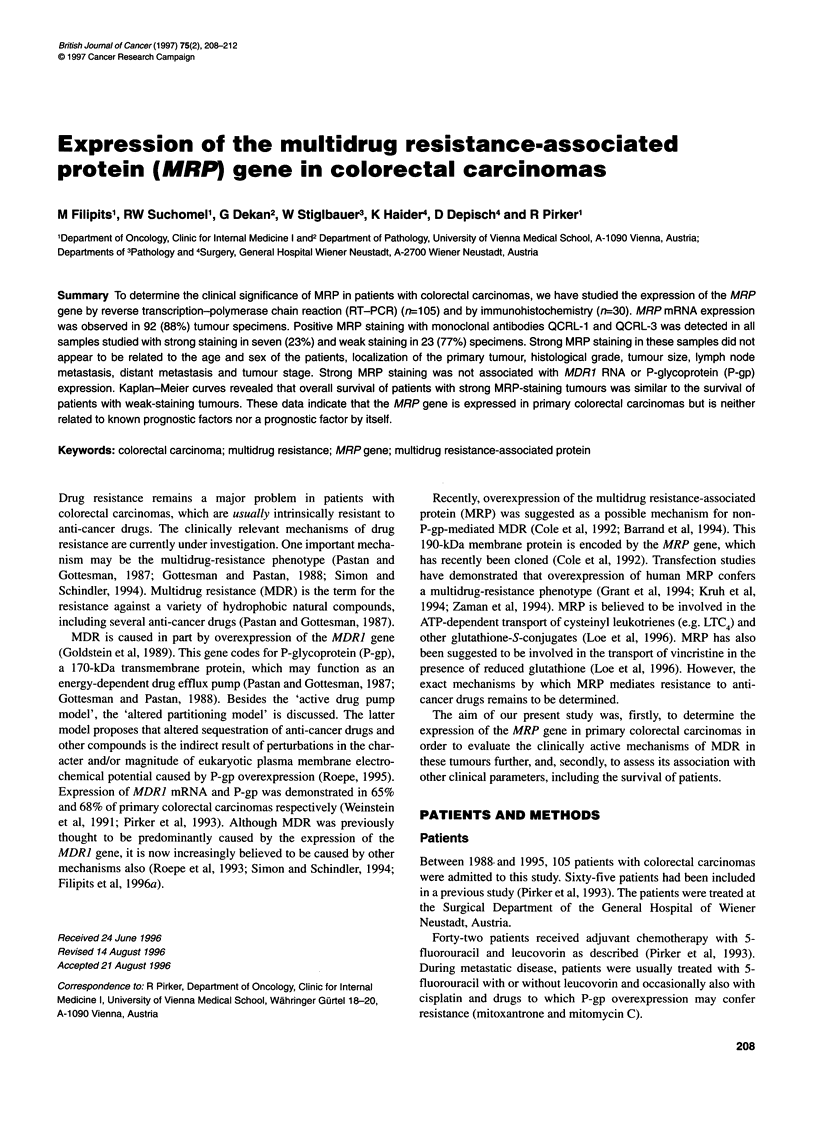

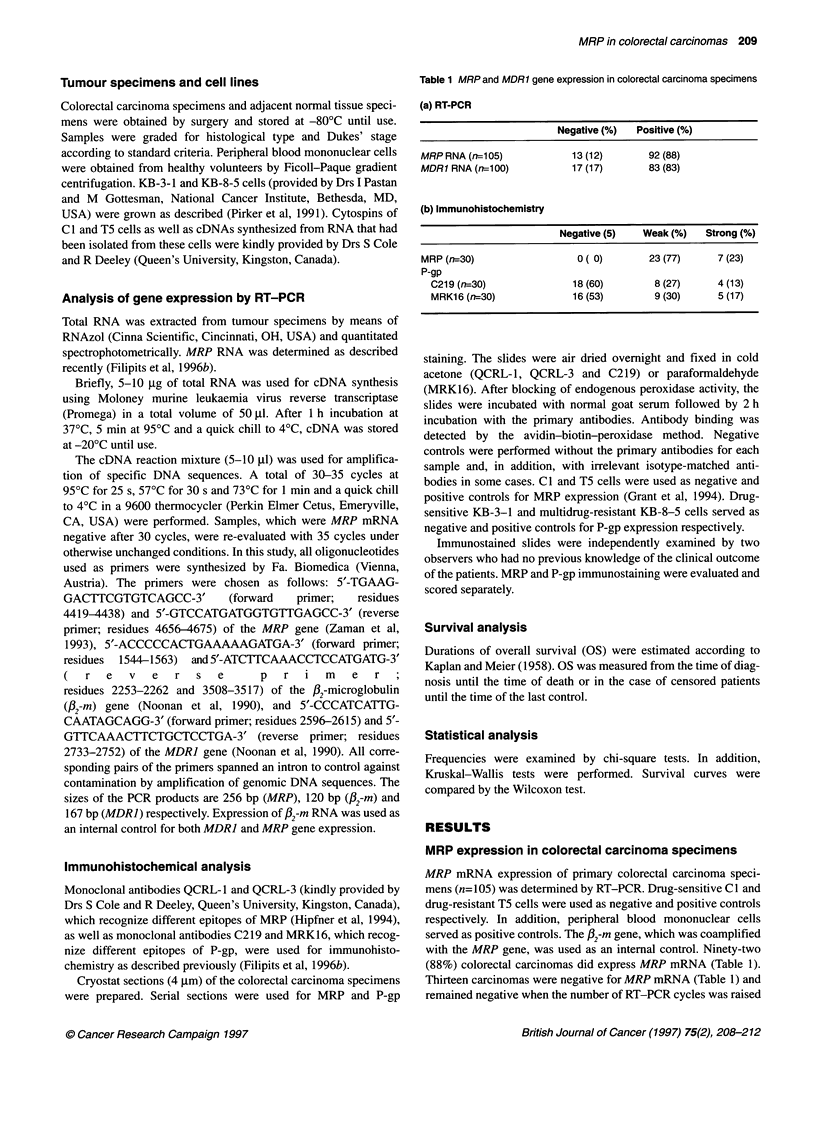

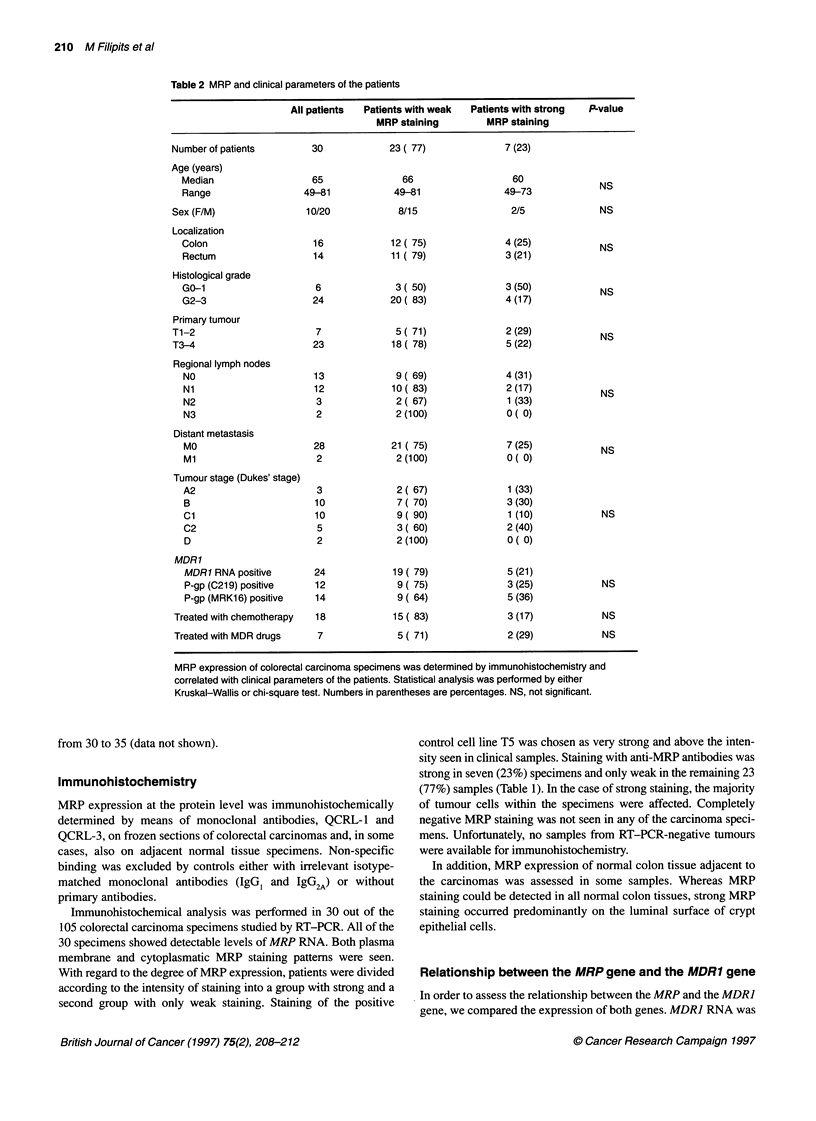

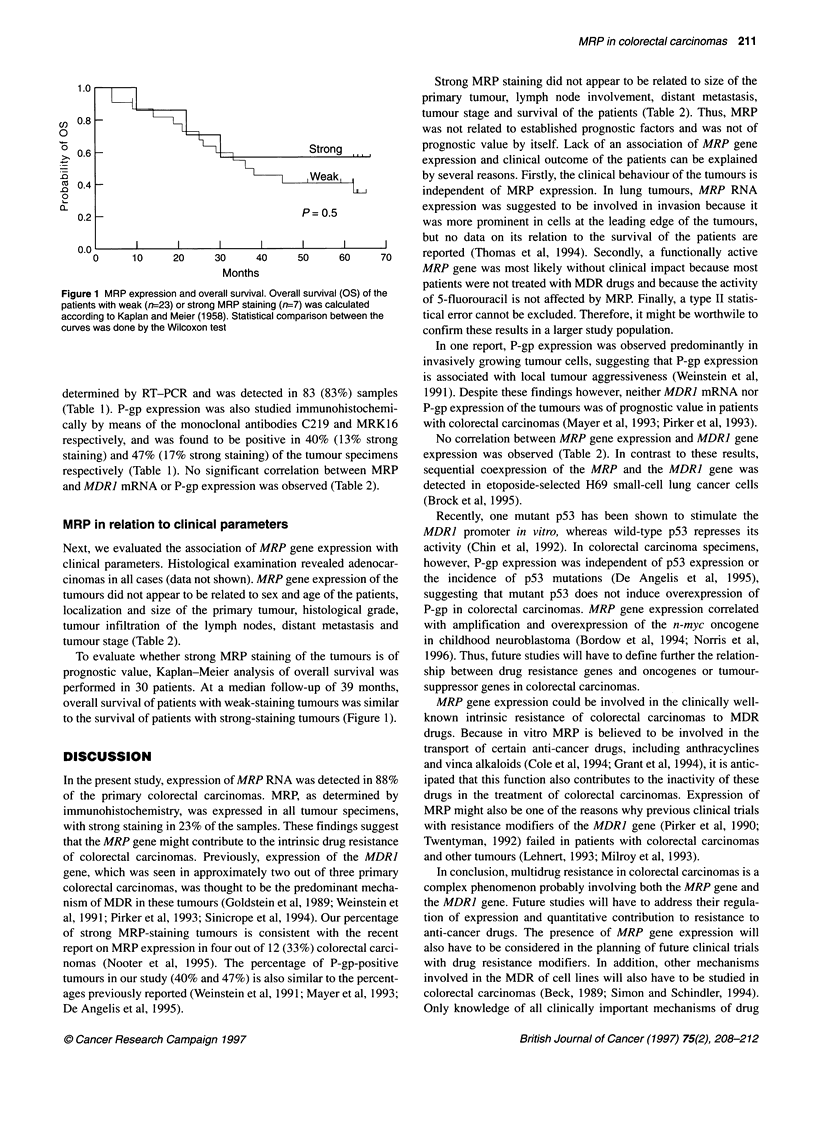

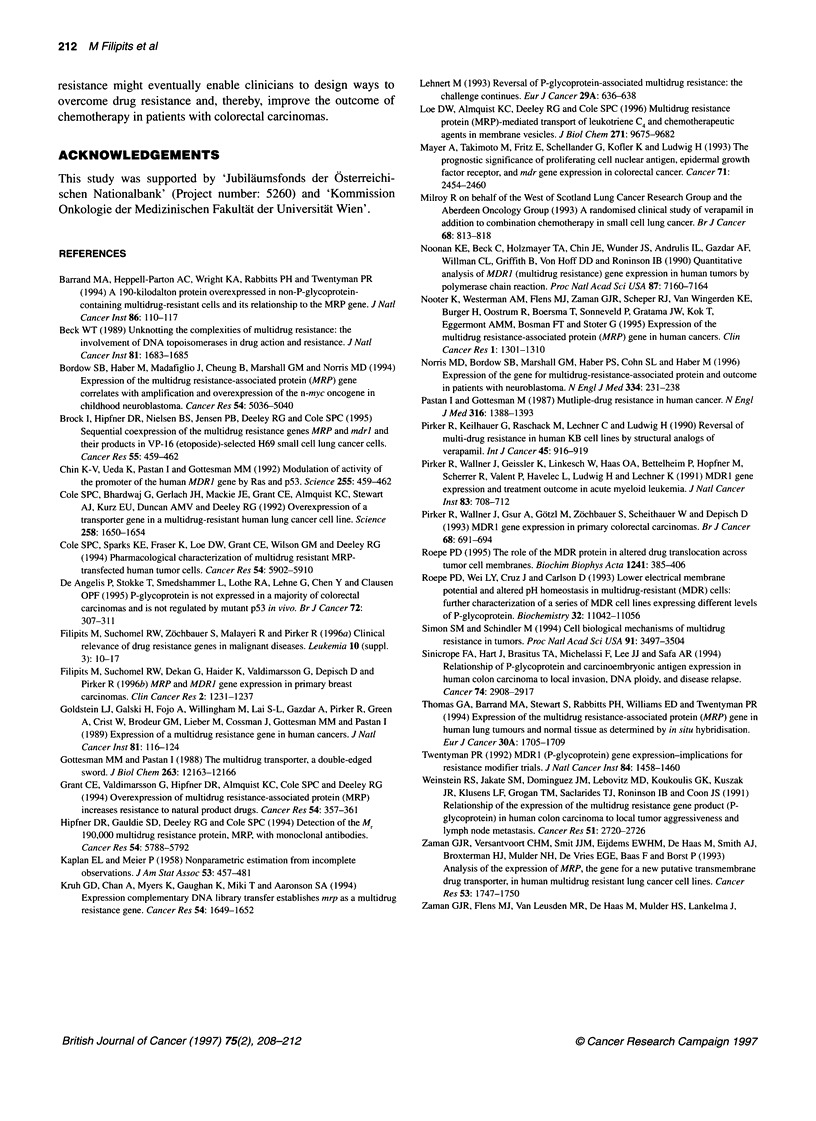


## References

[OCR_00499] Barrand M. A., Heppell-Parton A. C., Wright K. A., Rabbitts P. H., Twentyman P. R. (1994). A 190-kilodalton protein overexpressed in non-P-glycoprotein-containing multidrug-resistant cells and its relationship to the MRP gene.. J Natl Cancer Inst.

[OCR_00506] Beck W. T. (1989). Unknotting the complexities of multidrug resistance: the involvement of DNA topoisomerases in drug action and resistance.. J Natl Cancer Inst.

[OCR_00511] Bordow S. B., Haber M., Madafiglio J., Cheung B., Marshall G. M., Norris M. D. (1994). Expression of the multidrug resistance-associated protein (MRP) gene correlates with amplification and overexpression of the N-myc oncogene in childhood neuroblastoma.. Cancer Res.

[OCR_00518] Brock I., Hipfner D. R., Nielsen B. S., Jensen P. B., Deeley R. G., Cole S. P., Sehested M. (1995). Sequential coexpression of the multidrug resistance genes MRP and mdr1 and their products in VP-16 (etoposide)-selected H69 small cell lung cancer cells.. Cancer Res.

[OCR_00524] Chin K. V., Ueda K., Pastan I., Gottesman M. M. (1992). Modulation of activity of the promoter of the human MDR1 gene by Ras and p53.. Science.

[OCR_00527] Cole S. P., Bhardwaj G., Gerlach J. H., Mackie J. E., Grant C. E., Almquist K. C., Stewart A. J., Kurz E. U., Duncan A. M., Deeley R. G. (1992). Overexpression of a transporter gene in a multidrug-resistant human lung cancer cell line.. Science.

[OCR_00534] Cole S. P., Sparks K. E., Fraser K., Loe D. W., Grant C. E., Wilson G. M., Deeley R. G. (1994). Pharmacological characterization of multidrug resistant MRP-transfected human tumor cells.. Cancer Res.

[OCR_00539] De Angelis P., Stokke T., Smedshammer L., Lothe R. A., Lehne G., Chen Y., Clausen O. P. (1995). P-glycoprotein is not expressed in a majority of colorectal carcinomas and is not regulated by mutant p53 in vivo.. Br J Cancer.

[OCR_00551] Filipits M., Suchomel R. W., Dekan G., Haider K., Valdimarsson G., Depisch D., Pirker R. (1996). MRP and MDR1 gene expression in primary breast carcinomas.. Clin Cancer Res.

[OCR_00556] Goldstein L. J., Galski H., Fojo A., Willingham M., Lai S. L., Gazdar A., Pirker R., Green A., Crist W., Brodeur G. M. (1989). Expression of a multidrug resistance gene in human cancers.. J Natl Cancer Inst.

[OCR_00562] Gottesman M. M., Pastan I. (1988). The multidrug transporter, a double-edged sword.. J Biol Chem.

[OCR_00566] Grant C. E., Valdimarsson G., Hipfner D. R., Almquist K. C., Cole S. P., Deeley R. G. (1994). Overexpression of multidrug resistance-associated protein (MRP) increases resistance to natural product drugs.. Cancer Res.

[OCR_00571] Hipfner D. R., Gauldie S. D., Deeley R. G., Cole S. P. (1994). Detection of the M(r) 190,000 multidrug resistance protein, MRP, with monoclonal antibodies.. Cancer Res.

[OCR_00580] Kruh G. D., Chan A., Myers K., Gaughan K., Miki T., Aaronson S. A. (1994). Expression complementary DNA library transfer establishes mrp as a multidrug resistance gene.. Cancer Res.

[OCR_00585] Lehnert M. (1993). Reversal of P-glycoprotein-associated multidrug resistance: the challenge continues.. Eur J Cancer.

[OCR_00589] Loe D. W., Almquist K. C., Deeley R. G., Cole S. P. (1996). Multidrug resistance protein (MRP)-mediated transport of leukotriene C4 and chemotherapeutic agents in membrane vesicles. Demonstration of glutathione-dependent vincristine transport.. J Biol Chem.

[OCR_00594] Mayer A., Takimoto M., Fritz E., Schellander G., Kofler K., Ludwig H. (1993). The prognostic significance of proliferating cell nuclear antigen, epidermal growth factor receptor, and mdr gene expression in colorectal cancer.. Cancer.

[OCR_00600] Milroy R. (1993). A randomised clinical study of verapamil in addition to combination chemotherapy in small cell lung cancer. West of Scotland Lung Cancer Research Group, and the Aberdeen Oncology Group.. Br J Cancer.

[OCR_00606] Noonan K. E., Beck C., Holzmayer T. A., Chin J. E., Wunder J. S., Andrulis I. L., Gazdar A. F., Willman C. L., Griffith B., Von Hoff D. D. (1990). Quantitative analysis of MDR1 (multidrug resistance) gene expression in human tumors by polymerase chain reaction.. Proc Natl Acad Sci U S A.

[OCR_00613] Nooter K., Westerman A. M., Flens M. J., Zaman G. J., Scheper R. J., van Wingerden K. E., Burger H., Oostrum R., Boersma T., Sonneveld P. (1995). Expression of the multidrug resistance-associated protein (MRP) gene in human cancers.. Clin Cancer Res.

[OCR_00622] Norris M. D., Bordow S. B., Marshall G. M., Haber P. S., Cohn S. L., Haber M. (1996). Expression of the gene for multidrug-resistance-associated protein and outcome in patients with neuroblastoma.. N Engl J Med.

[OCR_00627] Pastan I., Gottesman M. (1987). Multiple-drug resistance in human cancer.. N Engl J Med.

[OCR_00631] Pirker R., Keilhauer G., Raschack M., Lechner C., Ludwig H. (1990). Reversal of multi-drug resistance in human KB cell lines by structural analogs of verapamil.. Int J Cancer.

[OCR_00636] Pirker R., Wallner J., Geissler K., Linkesch W., Haas O. A., Bettelheim P., Hopfner M., Scherrer R., Valent P., Havelec L. (1991). MDR1 gene expression and treatment outcome in acute myeloid leukemia.. J Natl Cancer Inst.

[OCR_00642] Pirker R., Wallner J., Gsur A., Götzl M., Zöchbauer S., Scheithauer W., Depisch D. (1993). MDR1 gene expression in primary colorectal carcinomas.. Br J Cancer.

[OCR_00647] Roepe P. D. (1995). The role of the MDR protein in altered drug translocation across tumor cell membranes.. Biochim Biophys Acta.

[OCR_00651] Roepe P. D., Wei L. Y., Cruz J., Carlson D. (1993). Lower electrical membrane potential and altered pHi homeostasis in multidrug-resistant (MDR) cells: further characterization of a series of MDR cell lines expressing different levels of P-glycoprotein.. Biochemistry.

[OCR_00658] Simon S. M., Schindler M. (1994). Cell biological mechanisms of multidrug resistance in tumors.. Proc Natl Acad Sci U S A.

[OCR_00662] Sinicrope F. A., Hart J., Brasitus T. A., Michelassi F., Lee J. J., Safa A. R. (1994). Relationship of P-glycoprotein and carcinoembryonic antigen expression in human colon carcinoma to local invasion, DNA ploidy, and disease relapse.. Cancer.

[OCR_00668] Thomas G. A., Barrand M. A., Stewart S., Rabbitts P. H., Williams E. D., Twentyman P. R. (1994). Expression of the multidrug resistance-associated protein (MRP) gene in human lung tumours and normal tissue as determined by in situ hybridisation.. Eur J Cancer.

[OCR_00674] Twentyman P. R. (1992). MDR1 (P-glycoprotein) gene expression--implications for resistance modifier trials.. J Natl Cancer Inst.

[OCR_00685] Zaman G. J., Versantvoort C. H., Smit J. J., Eijdems E. W., de Haas M., Smith A. J., Broxterman H. J., Mulder N. H., de Vries E. G., Baas F. (1993). Analysis of the expression of MRP, the gene for a new putative transmembrane drug transporter, in human multidrug resistant lung cancer cell lines.. Cancer Res.

